# Intratumoral heterogeneity, treatment response, and survival outcome of ER‐positive HER2‐positive breast cancer

**DOI:** 10.1002/cam4.5788

**Published:** 2023-03-19

**Authors:** Natsuki Teruya, Hiroaki Inoue, Rie Horii, Futoshi Akiyama, Takayuki Ueno, Shinji Ohno, Shunji Takahashi

**Affiliations:** ^1^ Breast Surgical Oncology, Breast Oncology Center Cancer Institute Hospital, Japanese Foundation for Cancer Research Tokyo Japan; ^2^ Department of Oncotherapeutic Medicine, Graduate School of Medicine Tohoku University Sendai Japan; ^3^ Department of Thoracic, Endocrine Surgery and Oncology Tokushima Graduate School of Biomedical Sciences Tokushima City Japan; ^4^ Department of Pathology Saitama Cancer Center Saitama Japan; ^5^ Department of Pathology Cancer Institute Hospital, Japanese Foundation for Cancer Research Tokyo Japan; ^6^ Department of Pathology Cancer Institute, Japanese Foundation for Cancer Research Tokyo Japan; ^7^ Breast Oncology Center Cancer Institute Hospital, Japanese Foundation for Cancer Research Tokyo Japan; ^8^ Department of Medical Oncology Cancer Institute Hospital, Japanese Foundation for Cancer Research Tokyo Japan

**Keywords:** ER+HER2+ breast cancer, heterogeneity, Ki67, pCR, prognosis, treatment response

## Abstract

**Background:**

ER+HER2+ breast cancer requires most types of systemic therapies perioperatively. However, treatment resistance is often experienced. The current study investigated the predictive and prognostic value of intratumoral heterogeneity and conventional clinicopathological factors in patients with ER+HER2+ breast cancer.

**Methods:**

This research included two patient cohorts with ER+HER2+ breast cancer. Cohort A included patients who underwent surgery without neoadjuvant chemotherapy (NAC). Cohort B comprised patients who received NAC followed by surgery. Intratumoral heterogeneity was assessed via ER and HER2 double staining, and the number of cells stained with different patterns of ER and HER2 was counted.

**Results:**

In total, 11 of 92 tumors in cohort A and four of 45 tumors in cohort B consisted exclusively of double‐positive (ER+ and HER2+) cells (homogeneous). The rest had different combinations of cells (heterogeneous). The pathological complete response (pCR) rates differed based on tumoral cell components but not intratumoral heterogeneity. The pCR rate of tumors with ER−HER2+ cells but without HER2− cells was higher than that of others (45.5% vs 4.3%; *p* = 0.0013). Low ER and PgR Allred scores indicated better pCR rates than high scores (*p* = 0.0005 and 0.024, respectively). Multivariate analysis showed that the ER Allred score and cell component of ER−HER2+ cells without HER2− cells were independent predictors of pCR (*p* = 0.0055 and 0.0081, respectively).

In cohort B, posttreatment Ki67, but not pCR, was a prognostic factor of DFS and OS (*p* = 0.028 and 0.017, respectively). The prognostic value of combined posttreatment Ki67 and pCR was superior to that of either alone. Combined pCR and posttreatment Ki67 had an independent prognostic value for DFS and OS (*p* = 0.0068 and 0.0101, respectively).

**Conclusions:**

In ER+HER2+ breast cancer, the presence of ER−HER2+ cells without HER2− cells was independently associated with pCR. Combined posttreatment Ki67 and pCR can be more precise in predicting prognosis than pCR alone.

## Graphical abstract

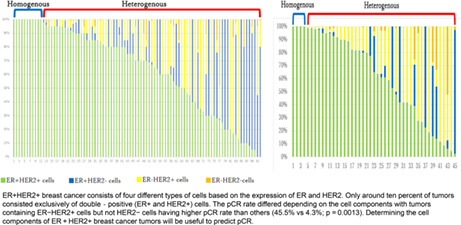

## 1 INTRODUCTION

The intrinsic subtypes of breast cancer based on gene expression profiling, as proposed by Perou et al., are correlated with treatment response and prognosis. Therefore, they are useful in determining appropriate treatment strategies.^1^, ^2^, ^3^, ^4^, ^5^ In clinical practice, immunohistochemistry‐based estrogen receptor (ER)/progesterone receptor (PgR)/human epidermal growth factor receptor 2 (HER2)/Ki67 examinations can be used to classify breast cancer based on four major subtypes, which are useful for treatment decision‐making.^6^, ^7^ ER+HER2+ breast cancer requires systemic therapies, such as chemotherapy, anti‐HER2 therapy, and endocrine therapy, perioperatively.^8^, ^9^, ^10^, ^11^, ^12^, ^13^ However, patients often experience treatment resistance.

In the NeoSphere and NeoALTTO trials, patients with HER2+ breast cancer received chemotherapy and anti‐HER2 therapy preoperatively. Results showed that the hormone receptor‐negative group had a higher pCR rate than the hormone receptor‐positive group.^14^, ^15^ Therefore, ER+HER2+ breast cancers are less sensitive to chemotherapy plus anti‐HER2 therapy than ER−HER2+ cancers.

The intratumoral heterogeneity, in which the histology of cancer differs spatially and temporally within the same tumor, can cause treatment resistance.^16^, ^17^ A comprehensive genomic analysis of cancer using next‐generation sequencing has shown the heterogeneity of cancer with genotypic and phenotypic diversity, which is associated with treatment resistance and cancer recurrence and metastasis.^16^, ^17^, ^18^, ^19^, ^20^, ^21^, ^22^, ^23^ ER + HER2+ tumors may contain mixed ER+ and HER2+ cancer cells at various proportions, which can cause intratumoral heterogeneity. We hypothesized that this can be a mechanism underlying treatment resistance.

The current study assessed intratumoral heterogeneity by counting single cells via ER and HER2 double staining. Further, the predictive and prognostic value of intratumoral heterogeneity and other conventional clinicopathological factors in patients with ER+HER2+ breast cancer was investigated.

## 2 PATIENTS AND METHODS

### 2.1 Patient cohorts

This is a retrospective observational study that included two patient cohorts with ER+HER2+ breast cancer. Cohort A included patients without neoadjuvant chemotherapy (NAC). The inclusion criteria were: unilateral invasive breast cancer that received surgical removal between January 2008 and December 2011 and ER+HER2+ breast cancer. The exclusion criteria were: preoperative systemic therapy administered and incisional biopsy performed at other hospitals. By reassessment of surgical specimen, no invasive cancer remaining, ER‐negative cancer or HER2‐negative cancer were also excluded. Cohort B comprised patients who received NAC. The inclusion criteria were: primary breast cancer that received surgery between January 2009 and December 2013, stage I‐III, HER2‐positive breast cancer, and NAC administered. The exclusion criteria were: needle biopsy performed at other hospitals and ER‐negative breast cancer.

### 2.2 Treatments

Patients with ER+HER2+ breast cancer were treated based on the National Comprehensive Cancer Network guidelines (Table S1).^24^

Neoadjuvant systemic therapy regimens comprised four cycles of CEF (cyclophosphamide 500 mg/m^2^, epirubicin 100 mg/m^2^, and 5‐fuorouracil 500 mg/m^2^, q3w) or six cycles of CAF (cyclophosphamide 500 mg/m^2^, adriamycin 50 mg/m^2^, and 5‐fuorouracil 500 mg/m^2^, q3w), followed by four cycles of tri‐weekly docetaxel at a dose of 75 mg/m^2^ or 12 cycles of weekly paclitaxel at a dose of 80 mg/m^2^. Trastuzumab was concurrently added to the taxane regimen. In adjuvant therapy, four cycles of AC (adriamycin 60 mg/m^2^, cyclophosphamide 600 mg/m^2^, q3w) or four cycles of AC, followed by taxane, were used.

### 2.3 Histopathological examination

We selected tissue blocks with the largest invasive cancer foci in the surgical specimen of patients in cohort A. ER, PgR, and HER2 immunohistochemistry, HER2 dual color in situ hybridization, and ER and HER2 double staining were performed. Table S2 shows the antibodies used. In ER/HER2 double staining, ER in the cell nucleus was detected as a red signal and HER2 in the cell membrane as a brown signal. This allowed the simultaneous evaluation of ER and HER2 in a single cell. We classified cancer cells into four types, which were as follows: ER+HER2− cells; ER+HER2+ cells; ER−HER2+ cells; and ER−HER2‐ cells. We calculated the percentage of each cell type by counting mostly at least 1000 cells. Biopsy specimens obtained prior to NAC and surgical specimens collected after NAC from patients in cohort B were used. Ki67 staining was performed based on the report of the International Working Group on Ki67 in Breast Cancer.^25^ The pathological response was assessed as grade 0–3 according to the histopathological criteria for the assessment of therapeutic response in breast cancer by the Japanese Breast Cancer Society (Table S3).^26^ pCR was defined as, regardless of the presence of in situ lesions, the disappearance of invasive cancer nests in the breast.

### 2.4 Follow‐up and prognosis

We collected data on the characteristics of patients, such as clinical findings, treatment, pathological factors, and posttreatment follow‐ups, from the medical records.

### 2.5 Statistical analysis

Differences in clinicopathological characteristics between the groups were assessed using the chi‐square test for categorical variables.

The Kaplan–Meier curves were obtained, and the log‐rank test was applied to compare the survival distributions of two populations. *p*‐values of <0.05 were considered statistically significant. All statistical analyses were performed using the JMP14 software (SAS Institute).

### 2.6 Ethical statement

This study was approved by the institutional review board and the ethical committee of the Japanese Foundation for Cancer Research (IRB‐2018‐1100). Patients' informed consent was waived by the ethical committee since this study was a retrospective investigation.

## 3 RESULTS

### 3.1 Heterogeneity in ER + HER2+ breast cancer

**FIGURE 1:**
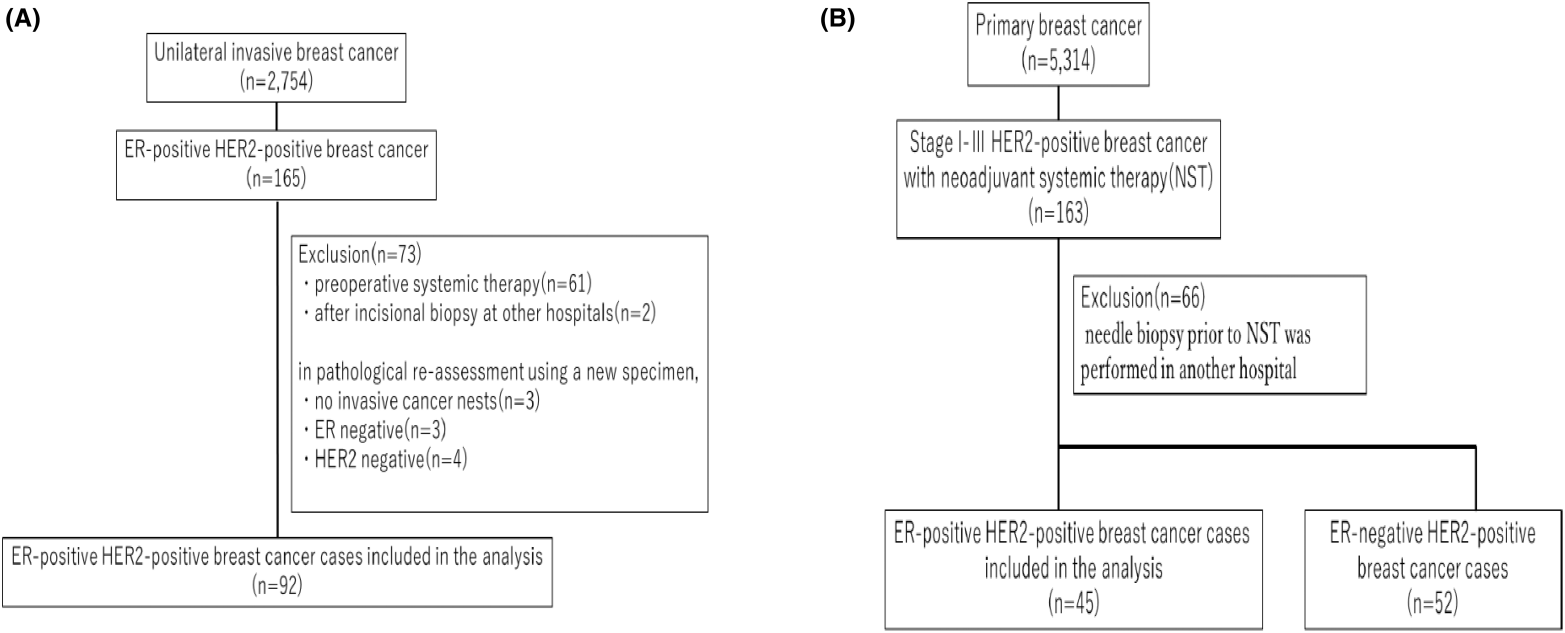
Flowchart of patient inclusion. (A) Cohort A. (B) Cohort B.

In total, 2754 patients had unilateral invasive breast cancer and received surgery between January 2008 and December 2011, of whom 165 had ER + HER2+ breast cancer. Out of these patients, 61 patients with preoperative systemic therapy, two patients who underwent incisional biopsy at other hospitals, three patients without evaluable invasive lesion in the surgical specimen, and three and four patients with ER− and HER2− cancer, respectively, on pathological reassessment were excluded. Hence, 92 patients were included in cohort A (Figure 1A). For cohort B, 5314 patients with primary breast cancer received surgery between January 2009 and December 2013, of whom 163 had stage I‐III, HER2‐positive breast cancer that received NAC. Out of these patients, 66 patients who underwent needle biopsy at other hospitals before NAC and 52 patients with ER− cancer were excluded. Thus, cohort B comprised 45 patients (Figure 1B).

**FIGURE 2:**
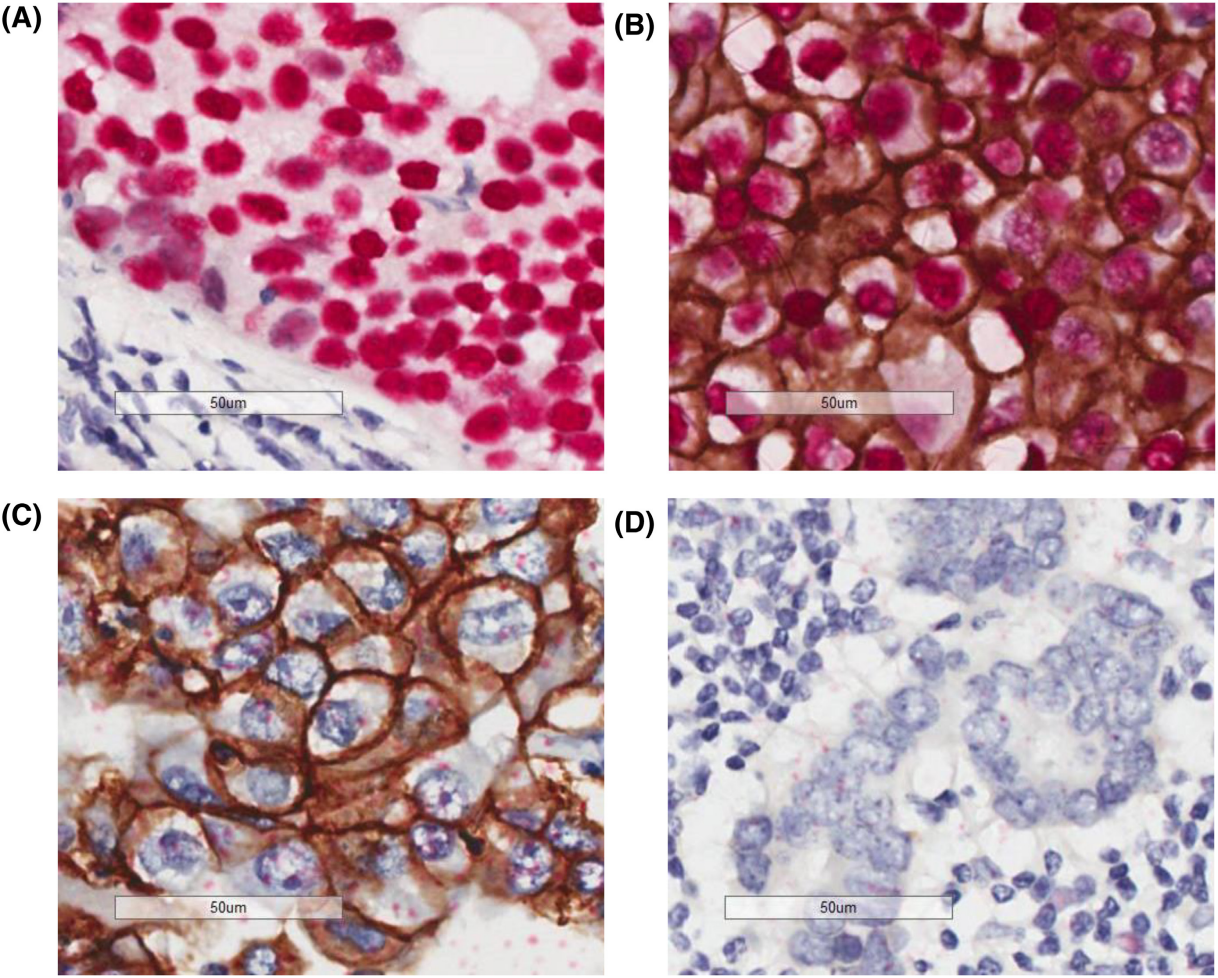
ER and HER2 double staining. ER in the nucleus is detected as a red signal and HER2 on the cell membrane is as a brown signal. (A) ER+ HER2− cells. (B) ER+ HER2+ cells. (C) ER− HER2+ cells. (D) ER−HER2− cells.

**FIGURE 3:**
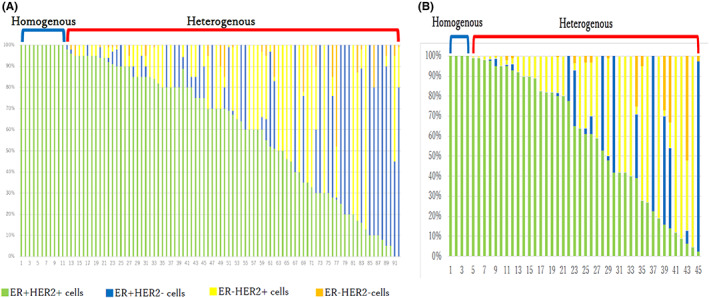
Proportion of each cell type in individual patients. (A) Cohort A. (B) Cohort B.

Intratumoral heterogeneity was assessed by examining the ER and HER2 status of a single cell in each specimen (Figure 2). Most tissues comprised at least two different types of cells (heterogenous tumors). Meanwhile, only 11 (12%) of 92 tissues in cohort A and 4 (8.9%) of 45 tissues in cohort B consisted exclusively of double‐positive (ER+ and HER2+) cells (homogeneous tumors) (Figure 3A,B). Two (2.2%) tumors in cohort A and none in cohort B did not have double‐positive cells. No significant differences in clinicopathological characteristics according to intratumoral heterogeneity were observed in cohort A (Table 1). Meanwhile, the clinical nodal status and axillary surgery types differed between the two groups in cohort B (Table 2).

**TABLE 1:** Clinicopathological characteristics by intratumoral heterogeneity in cohort A.

	Homogenous (%) (*n* = 11)	Heterogenous (%) (*n* = 81)	*p* value
Age			
Median (range)	54(45–67)	53 (26–82)	
Menopausal status			
Premenopausal	4 (36.4)	40 (49.4)	0.62
Postmenopausal	7 (63.6)	41 (50.6)	
Surgery type of breast			
Partial mastectomy	6 (54.5)	43 (53.1)	0.82
Mastectomy	5 (45.5)	38 (46.9)	
Surgery type of axilla			
Sentinel lymph node biopsy	8 (72.7)	50 (61.7)	0.71
Axillary dissection	3 (27.3)	31 (38.3)	
pT stage			
pT1	9 (81.8)	61 (75.3)	0.92
pT2	2 (18.2)	19 (23.5)	
pT3	0 (0)	1 (1.2)	
pN stage			
pN0	8 (72.7)	52 (64.2)	0.83
pN1	1 (9.1)	24 (29.6)	
pN2	1 (9.1)	4 (4.9)	
pN3	1 (9.1)	1 (1.2)	
pStage			
IA	7 (63.6)	42 (51.9)	0.68
IIA	3 (27.3)	24 (29.6)	
IIB	1 (9.1)	10 (12.3)	
IIIA	0 (0)	4 (4.9)	
IIIB	0 (0)	0 (0)	
IIIC	0 (0)	1 (1.2)	
Histological subtype			
Invasive ductal carcinoma	11 (100)	76 (93.8)	0.89
Invasive lobular carcinoma	0	2 (2.5)	
Other special type	0	3 (3.7)	
Nuclear grade			
1	1 (9.1)	6 (7.4)	0.68
2	6 (54.5)	38 (46.9)	
3	4 (36.4)	37 (45.7)	
PgR			
Negative	8 (72.7)	44 (54.3)	0.406
Positive	3 (27.3)	37 (45.7)	
HER2			
3+	10 (90.9)	52 (64.2)	0.15
2+	1 (9.1)	29 (35.8)	
Lymphatic invasion			
Negative	9 (81.8)	49 (60.5)	0.297
Positive	2 (18.2)	32 (39.5)	
Chemotherapy			
No	5 (45.5)	19 (23.5)	0.23
Yes	6 (54.5)	62 (76.5)	
Anti‐HER2 therapy			
No	5 (45.5)	18 (22.2)	0.28
Yes	6 (54.5)	61 (75.3)	
Unknown	0 (0)	2 (2.5)	
Endocrine therapy			
No	2 (18.2)	5 (6.2)	0.54
Yes	9 (81.8)	75 (92.6)	
Unknown	0 (0)	1 (1.2)	
Radiotherapy			
None	8 (72.7)	55 (67.9)	0.98
Conserved breast	2 (18.2)	20 (24.7)	
Chest wall and lymph node area	1 (9.1)	6 (7.4)	

**TABLE 2:** Clinicopathological characteristics in cohort B.

	Homogenous, (%) *n* = 4	Heterogeneity, *n* = 41 (%)	*p* value
Age			
Median (range)	55 (53–74)	53 (31–67)	
Menopausal status			
Premenopausal	0 (0)	17 (41.5)	0.274
Postmenopausal	4 (100)	24 (58.5)	
Clinical T category at diagnosis			
T1	1 (25)	5 (12.2)	0.959
T2	2 (50)	25 (61)	
T3	0 (0)	8 (19.5)	
T4	1 (25)	3 (7.3)	
Clinical N category at diagnosis			
N0	2 (50)	1 (2.4)	**0.00959**
N1	2 (50)	25 (61)	
N2	0 (0)	1 (2.4)	
N3	0 (0)	14 (34.1)	
Stage			
II	3 (75)	20 (48.8)	0.633
III	1 (25)	21 (51.2)	
Surgery type of breast			
Partial mastectomy	0 (0)	10 (24.4)	0.624
Mastectomy	4 (100)	31 (75.6)	
Surgery type of axilla			
Sentinel lymph node biopsy	2 (50)	1 (2.44)	**0.035**
Sampling	0 (0)	1 (2.44)	
Axillary dissection	2 (50)	39 (95.1)	
Histological subtype			
Invasive ductal carcinoma	4 (100)	39 (95.1)	0.413
Special type	0 (0)	2 (4.8)	
Nuclear grade			
1	2 (50)	13 (31.7)	0.865
2	1 (25)	10 (24.4)	
3	1 (25)	18 (43.9)	
PgR			
Negative	0 (0)	24 (58.5)	0.086
Positive	4 (100)	17 (41.5)	
HER2			
3+	4 (100)	32 (78)	0.694
2+	0 (0)	9 (22)	
Ki67 at baseline			
Median (range)	41.3 (27–49.4)	35.7 (4–80.3)	
Anti‐HER2 drug			
Yes	4 (100)	41 (100)	
Pathological tumor response Grade			
0	55 (53–74)	53 (31–67)	0.274
1a	0 (0)	17 (41.5)	
1b	4 (100)	24 (58.5)	
2a	1 (25)	5 (12.2)	
2b	2 (50)	25 (61)	
3	0 (0)	8 (19.5)	
Posttreatment Ki67			
Median (range)	6.3 (0–35.5)	5.8 (0–85.6)	

### 3.2 Predictive factor of pCR in ER + HER2+ breast cancer

The predictive value of intratumoral heterogeneity and the other clinicopathological factors of pCR was examined in cohort B. First, the predictive value of intratumoral heterogeneity for pCR was assessed. Because the statistical power was too low due to a small number in the homogeneous group, the statistical analysis comparing between homogeneous and non‐homogeneous groups was not performed but no clear association was observed between intratumoral heterogeneity and pCR. Next, we investigated the predictive value of each cell component according to ER and HER2 status (Table 3). The presence of ER − HER2+ cells was associated with a good pCR rate (30.6%). Meanwhile, the presence of ER + HER2− cells or ER − HER2− cells was correlated with poor pCR rates (5.3% for ER + HER2−, 7.1% for ER − HER2−). Thus, the presence of HER2− cells have poor pathological response. In fact, tumors with ER − HER2+ cells but without HER2− cells had a good pCR rate (45.5%, 10/22) (Table 4). Tables S4 and S5 depict the clinicopathological characteristics of cohorts A and B, respectively, according to cell component (presence of ER−HER2+ cells without HER2− cells). In cohort B, the presence of ER−HER2+ cells and the absence of HER2− cells were associated with a high pathological stage, negative PgR status, and HER2 protein expression of 3+ (Table S5).

**TABLE 3:** Cell component and pCR.

Cell component	pCR	non‐pCR	pCR rate (%)
ER + HER2+	11	34	24.4
ER + HER2−	1	18	5.3
ER − HER2+	11	25	30.6
ER − HER2−	1	13	7.1

**TABLE 4:** Clinicopathological factors and pCR.

	pCR	Non‐pCR	pCR rate	Chi square	Multivariate logistic regression	
				*p* value	Model A	Model B
Clinical T						
T1‐2	8	25	24.2%	0.96	—	—
T3‐4	3	9	25.0%			
Clinical N						
N0	0	3	0%	0.19	—	—
N1‐3	11	31	26.2%			
Stage						
II	4	19	17.4%	0.26	−	0.46
III	7	15	31.8			
ER Allred Score						
≥7	2	26	7.1%	**0.0005**	**0.0055**	**0.0071**
≤6	9	8	52.9%			
PgR Allred Score						
≥5	2	19	9.5%	**0.024**	0.11	0.12
≤4	9	15	37.5%			
HER2						
2+	1	8	11.1%	0.27	—	0.30
3+	10	26	27.8%			
NG						
1, 2	8	18	30.8%	0.24	—	—
3	3	16	15.8%			
NG						
1, 2	8	18	30.8%	0.24	—	—
3	3	16	15.8%			
Cell component						
ER−HER2+ without HER2−	10	12	45.5%	**0.0013**	**0.0081**	**0.0098**
Others	1	22	4.3%			

*Note*: Statistically significant *p* values are shown in bold.

Next, the association between conventional clinicopathological factors, including ER, PgR, HER2, and nuclear grade (NG), and pCR was examined. The cutoff values of ER and PgR Allred scores were set at 7 and 5, respectively, based on the Youden index via receiver operating characteristic curve analysis. As shown in Table 4, low ER and PgR Allred scores indicated better pCR rates. Meanwhile, HER2 expression and NG were not associated with pCR. Then, multivariate analyses were performed. In model A, factors with a *p* < 0.05 in the univariate analysis were included in the multivariate analysis. In model B, pathological stage and HER2 protein expression were further included to adjust for pathological stage, PgR status, and HER2 expression, which were associated with cell component in cohort B (Table S5). Logistic regression analysis showed that the ER Allred score and cell component were independent predictors of pCR in model A (*p* = 0.0055 and 0.0081, respectively). In model B, cell component of ER−HER2+ cells without HER2− cells were an independent predictor of pCR even after adjusting for pathological stage, PgR status, and HER2 protein expression (*p* = 0.0098).

### 3.3 Prognostic analysis of ER+ HER2+ breast cancer

**FIGURE 4:**
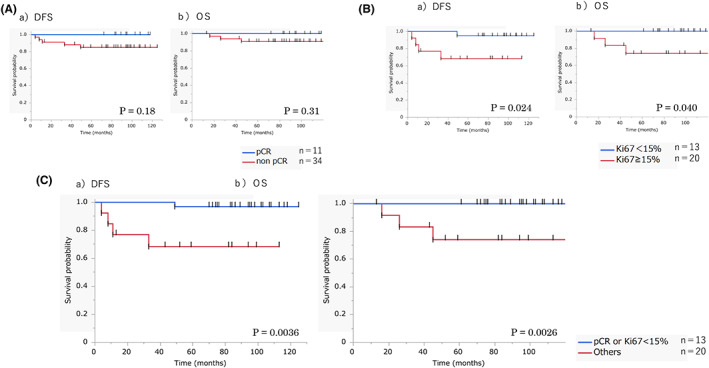
Survival analyses in cohort B. (A) Survival curves according to pathological response. (a) DFS: disease‐free survival, (b) OS: overall survival. (B) Survival curves according to posttreatment Ki67. (a) DFS: disease‐free survival, b) OS: overall survival. (C) Survival curves according to combined pathological response and posttreatment Ki67. (a) DFS: disease‐free survival, (b) OS: overall survival.

The prognostic values of clinicopathological factors in both cohorts were analyzed. In cohort A, none of the factors, including pT, pN, ER, PgR, HER2, NG, and cell component, had a prognostic significance on DFS or OS (Table 5). Because the statistical power was too low due to a small number in the homogeneous group, the statistical analysis comparing between homogeneous and non‐homogeneous groups was not performed. In cohort B, the aforementioned factors, including cell component, were not correlated with DFS. Meanwhile, cN and HER2 were associated with OS. In this cohort where patients received NAC, pCR was not associated with either DFS or OS. However, posttreatment Ki67 was a prognostic factor of both DFS and OS (Figure 4A,B, Table 5). If pCR and posttreatment Ki67 were combined, patients with either pCR or a posttreatment Ki67 of <15% had a significantly better DFS and OS than those without (*p* = 0.0036 and 0.0026, respectively; Figure 4C). Combined pCR and posttreatment Ki67 had a superior association with survival to either pCR or posttreatment Ki67 alone (Table 5). Factors with a *p* < 0.1 in the univariate analyses were included in the multivariate analysis in which ccombined pCR and posttreatment Ki67, but not posttreatment Ki67 alone, was included. Results showed that combined pCR and posttreatment Ki67 had an independent prognostic value for DFS and OS (*p* = 0.0068 and 0.0101, respectively).

**TABLE 5:** Prognostic analysis of ER‐positive HER2‐positive breast cancer.

		Univariate (log rank test)	
		DFS (*p* value)	OS (*p* value)
Cohort A			
pT	T1 vs T2‐3	0.71	0.94
pN	N0 vs N1‐3	0.86	0.47
ER	≥7 vs ≤6	0.50	0.70
PgR	≥5 vs ≤4	0.92	0.15
HER2	2+ vs 3+	0.81	0.72
NG	1, 2 vs 3	0.81	0.56
Cell component	ER− HER2+ without HER2− vs others	0.47	0.93

		Univariate (log rank test)		Multivariate (Cox regression)	
		DFS (*p* value)	OS (*p* value)	DFS (*p* value)	OS (*p* value)
Cohort B (NAC cohort)					
cT	T1‐2 vs T3‐4	0.065	0.105	0.073	—
cN	N0 vs N1‐3	0.13	**0.019**	—	0.058
ER	≥7 vs ≤6	0.43	0.95	—	—
PgR	≥5 vs ≤4	0.13	0.53	—	—
HER2	2+ vs 3+	0.17	**0.018**	—	**0.0221**
NG	1, 2 vs 3	0.36	0.33	—	—
Cell component	ER− HER2+ without HER2− vs others	0.17	0.078	—	1.0
pCR	pCR vs non‐pCR	0.18	0.31	—	−
Post Ki67	<15% vs others	**0.028**	**0.017**	—	−
pCR + post Ki67	pCR or post Ki67 < 15% vs others	**0.0036**	**0.0026**	**0.0068**	**0.0101**

## 4 DISCUSSION

The current study examined intratumoral heterogeneity via ER and HER2 double staining to simultaneously validate the expression of ER and HER2. Results showed that cell component, rather than intratumoral heterogeneity, affected response to NAC. Further, the presence of ER−HER2+ cells and the absence of HER2− cells indicated good pathological response even after adjusting for factors associated with cell component, which included pathological stage, PgR status, and HER2 protein expression. This result might be understandable considering that ER− tumors have higher pCR rates than ER+ tumors among patients with HER2+ breast cancers in clinical trials^15^ and that HER2+ tumors respond better to NAC (plus trastuzumab) than HER2− tumors.^27^ Currently, the omission of breast surgery after NAC is being tested for patients with good response to NAC in clinical trials.^28^ Therefore, determining the cell components of ER + HER2+ breast cancer tumors can be useful if surgical omission is applied after the validation of our study results.

Previous studies have reported an association between intratumoral heterogeneity and treatment response,^16^, ^17^, ^18^, ^19^, ^20^, ^21^, ^22^, ^23^ which was not shown in this study. Most of the previous studies used genetic analyses to see heterogeneity but this study used the expression of two markers, ER and HER2, and, thus, the biological meanings of heterogeneity can be different. In addition, the number in the homogeneous group was very small in this study, which made it difficult to make a reasonable comparison between homogeneous and heterogeneous groups. Further research is needed to clarify the clinical value of heterogeneity assessed by the method in this study.

In this study, pCR was not a prognostic factor of ER+HER2+ breast cancer, and posttreatment Ki67 was associated with DFS and OS. The prognostic impact of pCR differs based on breast cancer subtype. A meta‐analysis of HER2+ breast cancer showed that the difference in survival between patients with pCR and those without was greater in hormone receptor‐negative breast cancer than in hormone receptor‐positive breast cancer (hormone receptor‐negative: HR, 0.29 [95%PI, 0.24–0.36], hormone receptor‐positive: HR, 0.52 [95%PI, 0.4–0.66]).^29^ Another pooled analysis reported a similar result.^27^ This may explain, to some extent, our result showing that pCR had no prognostic impact in ER+HER2+ breast cancer, with consideration of our small sample size. Posttreatment Ki67 has an impact on survival.^30^ Furthermore, patients with hormone receptor‐positive breast cancer who achieved a posttreatment Ki67 of ≤15% has been reported to have a favorable DFS, which is comparable with that of patients who achieved pCR. However, this finding was not observed in patients with hormone receptor‐negative breast cancer.^31^ Thus, the prognostic impact of posttreatment Ki67 also differs based on breast cancer subtype, and it is greater in hormone receptor‐positive breast cancer. Our results showed that a combined analysis of posttreatment Ki67 and pCR could provide better surrogacy for survival than pCR alone in ER + HER2+ breast cancer. Thus, posttreatment Ki67 and pCR, rather than pCR alone, can achieve a more accurate prognostic prediction of ER + HER2+ breast cancer. Nevertheless, future clinical trials should be conducted to validate this notion.

The current study had several limitations. First, the sample size, particularly in cohort B, was small. Thus, our results, including those of the prognostic analyses, should be interpreted with caution. Second, the assessment of cell components was not simple, and hundreds of single cells should be counted cautiously. However, artificial intelligence has been making a significant advancement in image analyses and can be useful for the assessment of cell components in ER+HER2+ breast cancer.

## 5 CONCLUSION

In ER+HER2+ breast cancer, cell component influenced treatment response. That is, the presence of ER−HER2+ cells and the absence of HER2− cells were associated with a good pCR rate. Furthermore, combined posttreatment Ki67 and pCR can be more precise in predicting prognosis than pCR alone. However, a larger prospective study should be conducted to validate these results.

## AUTHOR CONTRIBUTIONS

**Natsuki Teruya:** Conceptualization (lead); data curation (lead); formal analysis (lead); investigation (equal); methodology (equal); resources (equal); software (equal); validation (equal); visualization (equal); writing – original draft (equal). **Hiroaki Inoue:** Data curation (equal); formal analysis (equal); investigation (equal); methodology (equal); software (equal). **Rie Horii:** Conceptualization (equal); data curation (equal); formal analysis (equal); methodology (equal); supervision (equal). **Futoshi Akiyama:** Conceptualization (equal); data curation (equal); supervision (equal). **Takayuki Ueno:** Conceptualization (equal); formal analysis (equal); writing – original draft (equal); writing – review and editing (equal). **Shinji Ohno:** Resources (equal); supervision (equal); writing – review and editing (equal). **Shunji Takahashi:** Conceptualization (equal); resources (equal); supervision (equal); writing – review and editing (equal).

## ETHICS STATEMENT

This study was approved by the ethical committee of the Japanese Foundation for Cancer Research (IRB‐2018‐1100). Patients' informed consent was waived by the ethical committee since this study was a retrospective investigation.

## Supporting information


Data S1.

## Data Availability

The datasets used and analyzed during the current study are available from the corresponding author on reasonable request.

## References

[1] Perou CM , Sørlie T , Michael B , et al. Molecular portraits of human breast tumors. Nature. 2000;406:747‐752. 10963602 10.1038/35021093

[2] Koboldt DC , Fulton R , Mclellan MD , et al. Comprehensive molecular portraits of human breast tumours. Nature. 2012;490(7418):61‐70. 23000897 10.1038/nature11412 PMC3465532

[3] Sørlie T , Perou CM , Tibshirani R , et al. Gene expression patterns of breast carcinomas distinguish tumor subclasses with clinical implications. PNAS. 2001;98(19):10869‐10874. 11553815 10.1073/pnas.191367098 PMC58566

[4] Goldhirsch A , Wood WC , Coates AS , et al. Strategies for subtypes–dealing with the diversity of breast cancer: highlights of the St Gallen international expert consensus on the primary therapy of early breast cancer 2011. Ann Oncol. 2011;22(8):1736‐1747. 21709140 10.1093/annonc/mdr304 PMC3144634

[5] Goldhirsch A , Winer EP , Coates AS , et al. Personalizing the treatment of women with early breast cancer: highlights of the St Gallen international expert consensus on the primary therapy of early breast cancer 2013. Ann Oncol. 2013;24(9):2206‐2223. 23917950 10.1093/annonc/mdt303 PMC3755334

[6] The Japanese Breast Cancer Society clinical practice guidelines for pathological diagnosis of breast cancer, 2015. Kanehara Publishing Co. 2015 edition; 225–226.

[7] Curigliano G , Burstein HJ , Winer EP , et al. De‐escalating and escalating treatments for early‐stage breast cancer: the St Gallen international expert consensus on the primary therapy of early breast cancer 2017. Ann Oncol. 2017;28(8):1700‐1712. 28838210 10.1093/annonc/mdx308 PMC6246241

[8] Smith I , Procter M , Gelber RD , et al. 2‐year follow‐up of trastuzumab after adjuvant chemotherapy in HER2‐positive breast cancer: a randomised controlled trial. Lancet. 2007;369(9555):29‐36. 17208639 10.1016/S0140-6736(07)60028-2

[9] Gianni L , Dafni U , Gelber RD , et al. Treatment with trastuzumab for 1 year after adjuvant chemotherapy in patients with HER2‐positive early breast cancer: a 4‐year follow‐up of a randomised controlled trial. Lancet Oncol. 2011;12(3):236‐244. 21354370 10.1016/S1470-2045(11)70033-X

[10] Goldhirsch A , Gelber RD , Piccart‐Gebhart MJ , et al. 2 years versus 1 year of adjuvant trastuzumab for HER2‐positive breast cancer (HERA): an open‐label, randomised controlled trial. Lancet. 2013;382(9897):1021‐1028. 23871490 10.1016/S0140-6736(13)61094-6

[11] Romond EH , Perez EA , Bryant J , et al. Trastuzumab plus adjuvant chemotherapy for operable HER2‐positive breast cancer. N Engl J Med. 2005;353(16):1673‐1684. 16236738 10.1056/NEJMoa052122

[12] Perez EA , Suman VJ , Davidson NE , et al. Sequential versus concurrent trastuzumab in adjuvant chemotherapy for breast cancer. J Clin Oncol. 2011;29(34):4491‐4497. 22042958 10.1200/JCO.2011.36.7045 PMC3236650

[13] Slamon D , Ei ermann W , Robert N , et al. Adjuvant Trastuzumab in HER2‐positive breast cancer. N Engl J Med. 2011;365(14):1273‐1283. 21991949 10.1056/NEJMoa0910383 PMC3268553

[14] Gianni L , Pienkowski T , Im YH , Roman L , et al. Efficacy and safety of neoadjuvant pertuzumab and trastuzumab in women with locally advanced, inflammatory, or early HER2‐positive breast cancer (NeoSphere): a randomised multicentre, open‐label, phase 2 trial. Lancet Oncol. 2012;13(1):25‐32. 22153890 10.1016/S1470-2045(11)70336-9

[15] Baselga J , Btadbury I , Eidtmann H , et al. Lapatinib with trastuzumab for HER2‐positive early breast cancer (NeoALTTO): a randomised, open‐label, multicentre, phase 3 trial. Lancet. 2012;379(9816):633‐640. 22257673 10.1016/S0140-6736(11)61847-3 PMC5705192

[16] Martelotto LG , Ng CKY , Piscuoglio S , Weigelt B , Reis‐Filho JS . Breast cancer intra‐tumor heterogeneity. Breast Cancer Res. 2014;16(3):R48. 10.1186/bcr3658 PMC4053234 25928070

[17] Zardavas D , Irrthum A , Swanton C , Piccart M . Clinical management of breast cancer heterogeneity. Nat Rev Clin Oncol. 2015;12(7):381‐394. 25895611 10.1038/nrclinonc.2015.73

[18] Vogelstein B , Papadopoulos N , Velculescu VE , Zhou S , Diaz LA , Kinzler KW . Cancer genome landscapes. Science. 2013;339(6127):1546‐1558. 23539594 10.1126/science.1235122 PMC3749880

[19] Gerlinger M , Rowan AJ , Horswell S , et al. Intratumor heterogeneity and branched evolution revealed by multiregion sequencing. N Engl J Med. 2012;366(10):883‐892. 22397650 10.1056/NEJMoa1113205 PMC4878653

[20] Yates LR , Gerstung M , Knappskog S , et al. Subclonal diversification of primary breast cancer revealed by multiregion sequencing. Nat Med. 2015;21(7):751‐759. 26099045 10.1038/nm.3886 PMC4500826

[21] McGranahan N , Swanton C . Clonal heterogeneity and tumor evolution:past, present, and the future. Cell. 2017;168:613‐628. 28187284 10.1016/j.cell.2017.01.018

[22] Yates LR , Gerstung M , Knappskog S , et al. Subclonal diversification of primary breast cancer revealed by multiregion sequencing. Nat Med. 2015; 21(7):751‐7591 26099045 10.1038/nm.3886 PMC4500826

[23] Lee HJ , Kim JY , Park SY , et al. Clinicopathologic significance of the Intratumoral heterogeneity of HER2 gene amplification in HER2‐ positive breast cancer patients treated with adjuvant Trastuzumab. Am J Clin Pathol. 2015;144:570‐578. 26386078 10.1309/AJCP51HCGPOPWSCY

[24] NCCN (the National Comprehensive Cancer Network) guideline Ver6 . (2020). https://www.nccn.org/professionals/physician_gls/pdf/breast.pdf

[25] Nielsen TO , Leung SCY , Rimm DL , et al. Assessment of Ki67 in breast cancer:updated recommendations from the International Ki67 in Breast Cancer Working Group. J Natl Cancer Inst. 2021;113(7):808‐819. 33369635 10.1093/jnci/djaa201 PMC8487652

[26] Japanese Brease Cancer Society . General rules for clinical and pathological recording if breast cancer. 17th ed. Japanese Breast Cancer Society; 2012:84.

[27] Cortazar P , Zhang L , Untch M , et al. Pathological complete response and long‐term clinical benefit in breast cancer: the CTNeoBC pooled analysis. Lancet. 2014;384:164‐172. 24529560 10.1016/S0140-6736(13)62422-8

[28] Shigematsu H , Fujisawa T , Shien T , Iwata H . Omitting surgery for early breast cancer showing clinical complete response to primary systemic therapy. JJCO. 2020;50:629‐634. 32378709 10.1093/jjco/hyaa055

[29] Broglio KR , Quintana M , Foster M , et al. Association of pathologic complete response to neoadjuvant therapy in HER2‐positive breast cancer with long‐term outcomes: a meta‐analysis. JAMA Oncol. 2016;2(6):751‐760. 26914222 10.1001/jamaoncol.2015.6113

[30] Jones RL , Salter J , A'Hern R , et al. The prognostic significance of Ki67 before and after neoadjuvant chemotherapy in breast cancer. Breast Cancer Res Treat. 2009;116(1):53‐68. 18592370 10.1007/s10549-008-0081-7

[31] von Minckwitz G , Schmitt WD , Loibi S , et al. Ki67 measured after neoadjuvant chemotherapy for primary breast cancer. Clin Cancer Res. 2013;19:4521‐4531. 23812670 10.1158/1078-0432.CCR-12-3628

