# Supplementary material for: Intratumoral heterogeneity, treatment response, and survival outcome of ER‐positive HER2‐positive breast cancer
Source: Cancer Med. 2023 Mar 19;12(9):10526–35. doi: 10.1002/cam4.5788 (PMC10225233; doi:10.1002/cam4.5788)
Supplement: Supplementary file 1 — Data S1. [file CAM4-12-10526-s001.docx]

Supplementary Table 1 Perioperative therapy for ER+HER2+ breast cancer

|  |  | Diameter of invasion（T）㎝ | Indications for drug therapy |
| --- | --- | --- | --- |
| Lymph node metastasis | negative | T≦0.5 | ①none  ②endocrine therapy |
|  |  | 0.5<T≦2.0 | Chemotherapy, anti-HER2 therapy, endocrine therapy  Chemotherapy is selected from the following options, taking into account age and other risks  ①taxane alone  ➁anthracyclin alone  ③anthracyclin followed by taxane |
|  |  | 2.0<T | Chemotherapy, anti-HER2 therapy*, endocrine therapy  ①anthracyclin followed by taxane  ➁anthracyclin alone |
|  | positive | any T | Chemotherapy, anti-HER2 therapy*, endocrine therapy |

＊The combination of trastuzumab and pertuzumab is currently indicated as anti-HER2 therapy for patients at high risk of recurrence, but only trastuzumab was administered as anti-HER2 therapy during the period covered in this study.

Supplementary Table 2 List of antibodies used in this study

|  | January 2008 to February 2012 | March 2012 to December 2013 |
| --- | --- | --- |
| ER | clone 1D5, (Dako Japan Inc., Tokyo, Japan) | Clone ERSP1, (Roche Diagnostics, Tokyo, Japan) |
| PgR | clone PgR636, (Dako Japan Inc.) | ClonePgR1E2, (Roche Diagnostics) |
| HER2 | Hercep Test (Dako Japan Inc.) | HER2 antibody (HER2 4B5, Roche Diagnostics) |
| HER2 FISH | PathVysion HER-2 DNA Probe Kit (Abbott Molecular Inc.,Des Plaines, IL, USA) | - |
| HER2 DISH | - | INFORM HER2 Dual ISH DNA Probe Cocktail (Roche Diagnostics) |
| Ki67 | MIB-1, (DAKO, Denmark), | |
| Double staining  ER  HER2 | Clone ERSP1, (Roche Diagnostics, Tokyo, Japan)  HER2 antibody (HER2 4B5, Roche Diagnostics) | |

Supplementary Table 3 Histopathological criteria for assessment of therapeutic response

| Grade | |  |
| --- | --- | --- |
| 0　 no response | | Almost no change in invasive cancer cells after treatment |
| 1　slight response | a）mild response | Mild changes in invasive cancer cells regardless of the area, or marked changes are seen in less than one-third of cancer cells |
|  | b）moderate response | Marked changes in one-thirds or more of invasive cancer cells |
| 2　marked response | a）marked response | Marked changes in two-thirds or more of invasive cancer cells |
|  | b）extremely marked response | Less than a few clusters of invasive cancer cells remaining |
| 3　complete response | | Necrosis or disappearance of all invasive cancer cells; replacement of all cancer cells by granuloma-like and/or fibrous tissue |

The general rules for clinical and pathological recording of breast cancer(17^th^ edition)

Supplementary Table 4 Clinicopathologic characteristics by cell component of ER−HER2+ cells without HER2− cells in cohort A

|  |  | Cell component of ER−HER2+ without  HER2− cells  n＝26（％） | Others  n＝66（％） | *P* value |
| --- | --- | --- | --- | --- |
| Age | Median (range) | 53 (38-62） | 53 (26-82） |  |
| Menopausal status | Premenopausal | 15 (57.7） | 29 (43.9) | 0.23 |
|  | Postmenopausal | 11(42.3) | 37 (56.1) |  |
| Surgery type of breast | Partial mastectomy | 13 (50) | 36 (54.6) | 0.69 |
|  | mastectomy | 13 (50) | 30 (45.5) |  |
| Surgery type of axilla | Sentinel lymph node biopsy | 18 (69.2) | 43 (65.2) | 0.71 |
|  | Axillary dissection | 8 (30.8) | 23 (34.9) |  |
| pT stage | pT1 | 21 (80.7) | 49 (74.2) | 0.50 |
|  | pT2 | 4 (15.4) | 17 (25.8) |  |
|  | pT3 | 1 (3.9) | 0 (0) |  |
| pN stage | pN0 | 19 (73.1) | 41 (62.1) | 0.50 |
|  | pN1 | 4 (15.4) | 23 (34.9) |  |
|  | pN2 | 2 (7.7) | 2 (3.0) |  |
|  | pN3 | 1 (3.9) | 0 (0) |  |
| pStage | IA | 16 (61.5) | 33 (50) | 0.21 |
|  | ⅡA | 5 (19.2) | 22 (33.3) |  |
|  | ⅡB | 2 (7.7) | 9 (13.6) |  |
|  | ⅢA | 2 (7.7) | 2 (3.0) |  |
|  | ⅢB | 0 (0) | 0 (0) |  |
|  | ⅢC | 1 (3.9) | 0 (0) |  |
| Histological subtype | Invasive ductal carcinoma | 24 (92.3) | 63 (95.5) | 0.88 |
|  | Invasive lobular carcinoma | 1 (3.9) | 1 (1.5) |  |
|  | Other special type | 1 (3.9) | 2 (3.0) |  |
| Nuclear grade | 1 | 3 (11.5) | 4 (6.1) | 0.45 |
|  | 2 | 10 (38.5) | 34 (51.5) |  |
|  | 3 | 13 (50) | 28 (42.4) |  |
| PgR | Negative | 17 (65.4) | 35 (53) | 0.28 |
|  | Positive | 9 (34.6) | 31 (47) |  |
| HER2 | 3+ | 24 (92.3) | 38 (57.6) | 0.0005 |
|  | 2+ | 2 (7.7) | 28 (42.4) |  |
| Lymphatic invasion | Negative | 16 (61.5) | 42 (63.6) | 0.85 |
|  | Positive | 10 (38.5) | 24 (36.4) |  |
| Chemotherapy | No | 8 (30.8) | 16 (24.2) | 0.52 |
|  | Yes | 18 (69.2) | 50 (75.8) |  |
| Anti-HER2 therapy | No | 7 (26.9) | 16 (24.2) | 0.50 |
|  | Yes | 19 (73.1) | 48 (72.7) |  |
|  | Unknown | 0 (0) | 2 (3.0) |  |
| Endocrine therapy | No | 2 (7.7) | 5 (7.6) | 0.55 |
|  | Yes | 24 (92.3) | 60 (90.9) |  |
|  | Unknown | 0 (0) | 1 (1.5) |  |
| Radiotherapy | None | 19 (73.1) | 44 (66.7) | 0.55 |
|  | Conserved breast | 5 (19.2) | 17 (25.8) |  |
|  | Chest wall and lymph node area | 2 (7.7) | 5 (7.6) |  |

Supplementary Table 5 Clinicopathologic characteristics by cell component of ER−HER2+ cells without HER2− cells in cohort B

|  |  | Cell component of ER−HER2+ without  HER2− cells  n＝22（％） | Others  n＝23（％） | *P* value |
| --- | --- | --- | --- | --- |
| Age | Median (range) |  |  |  |
| Menopausal status | Premenopausal | 10 (45.5) | 7 (30.4) | 0.30 |
|  | Postmenopausal | 12 (54.5) | 16 (69.6) |  |
| Clinical T  category at diagnosis | T1 | 3 (13.6) | 3 (13) | 0.70 |
|  | T2 | 11 (50) | 16 (69.6) |  |
|  | T3 | 8 (36.4) | 0 (0) |  |
|  | T4 | 0 (0) | 4 (17.4) |  |
| Clinical N  category at diagnosis | N0 | 0 (0) | 3 (13) | 0.25 |
|  | N1 | 12 (54.6) | 15 (65.2) |  |
|  | N2 | 1 (4.6) | 0 (0) |  |
|  | N3 | 9 (40.9) | 5 (21.7) |  |
| Stage | II | 7 (31.8) | 16 (69.6) | **0.01** |
|  | III | 15 (68.2) | 7 (30.4) |  |
| Surgery type of breast | Partial mastectomy | 4 (18.2) | 6 (26.1) | 0.52 |
|  | mastectomy | 18 (81.8) | 17 (73.9) |  |
| Surgery type of axilla | Sentinel lymph node biopsy | 0 | 3 (13) | 0.06 |
|  | Sampling | 0 | 1 (4.4) |  |
|  | Axillary dissection | 22 (100) | 19 (82.6) |  |
| Histological subtype | Invasive ductal carcinoma | 19 (86.4) | 22 (95.7) | 0.12 |
|  | Special type | 3 (13.6) | 1 (4.3) |  |
| Nuclear grade | 1 | 7 (31.8) | 8 (34.8) | 0.91 |
|  | 2 | 5 (22.7) | 6 (26.1) |  |
|  | 3 | 10 (45.5) | 9 (39.1) |  |
| PgR | Negative | 16 (72.7) | 8 (34.8) | **0.0097** |
|  | Positive | 6 (27.3) | 15 (65.2) |  |
| HER2 | 3+ | 22 (100) | 14 (60.9) | **0.0036** |
|  | 2+ | 0 (0) | 9 (39.1) |  |
| Ki67 | median (range) | 37.7  (15.8-53.9) | 35.7  (4-80.3) |  |
| Anti-HER2 drug | Yes | 22 (100) | 23 (100) |  |
| Pathological tumor response  Grade | 0 | 0 (0) | 0 (0) |  |
|  | 1a | 1 (4.6) | 6 (26.1) |  |
|  | 1b | 0 (0) | 4 (17.4) |  |
|  | 2a | 8 (36.4) | 10 (43.5) |  |
|  | 2b | 3 (13.6) | 2 (8.7) |  |
|  | 3 (pCR) | 10 (45.5) | 1 (4.4) | **0.0042** |
| Posttreatment Ki67 | median (range) | 5.8 (0-46.5) | 6.3 (0-85.6) |  |
